# Sweep Pulse Excitation Method for Enhancing Photoacoustic Elastic Waves at Different Laser Irradiation Parameters

**DOI:** 10.3390/s22135025

**Published:** 2022-07-03

**Authors:** Katsuhiro Mikami, Natsumi Sudo, Yuka Okamoto, Takeo Nagura, Daisuke Nakashima

**Affiliations:** 1 Faculty of Biology-Oriented Science and Technology, Kindai University, Wakayama 649-6493, Japan; wisterias3719@gmail.com (N.S.); yokamoto199956@gmail.com (Y.O.); 2 Department of Orthopedic Surgery, Keio University School of Medicine, Tokyo 160-8582, Japan; nagura@keio.jp (T.N.); nakashima@keio.jp (D.N.); 3 Department of Clinical Biomechanics, Keio University School of Medicine, Tokyo 160-8582, Japan

**Keywords:** laser remote sensing, sweep pulse excitation, photoacoustic elastic wave, resonance effect, spatiotemporal dynamics, vibrational excitation dynamics

## Abstract

Laser remote sensing using a sweep pulse excitation method, in which a laser beam is irradiated at the same repetition frequency as the natural frequency, for enhancing photoacoustic elastic waves through resonance effect has been studied. The sweep pulse excitation method, which is based on the principle of detecting natural frequency fluctuations, such as hammering tests, can detect natural frequencies in the audible sound region with low average laser power and contribute to the convenience and low cost of an installation strength diagnosis of fastening bolts. In this study, we investigated the dynamics of the swept excitation method for optimization by evaluating the dependence of the laser irradiation conditions (pulse width, spot size, and average power) on different metal disc samples. We discovered that the magnitude of the photoacoustic elastic wave is proportional to the absorption of laser power, and the spatiotemporal dynamics can be explained through thermal diffusion phenomena. These findings contribute to the development of laser-sensing technology based on photoacoustic elastic waves.

## 1. Introduction

The vibrations induced by laser irradiation due to thermal expansion and/or ablation can present the optical information and mechanical information of the evaluated sample and constitute one of the fundamental principles for laser remote sensing (LRS). For instance, nanosecond order pulses are an excellent source of impulse excitation. LRS for measuring vibrations in the ultrasonic band has been reported previously [1]. LRS has been applied to biological measurements, such as photoacoustic imaging (PAI), which enables measurements of blood capillaries on the skin with a high spatial resolution [2,3,4]. Additionally, PAI for observing living cells has also been demonstrated previously [5]. Specifically, LRS that measures vibrations in the audible sound range has been studied. Recently, high-speed quantitative inspections [6,7] of the internal defects in concretes and bolt fastening [8] have been reported. LRS aims at replacing the hammering inspection performed by inspectors using laser technology. In LRS, within the audible frequency range, the vibrations induced by laser ablation are used to measure the natural vibrations of an irradiated sample [9,10]. During recent years, medical techniques applying LRS, such as laser resonance frequency analysis (L-RFA), have been reported, particularly in the field of orthopedics, where implant stability diagnostics have been developed to prevent implant failures. The natural frequency observed in the diagnosis, including the installation interface condition between the orthopedic implant and the base material (such as bone), has been correlated with the mechanical strength of the installation reference measured at the laboratory level [11,12,13,14]. Moreover, a medical diagnostic technique using RFA has been proposed; this method utilizes magnetic pulses as the excitation source (magnetic resonance frequency analysis: M-RFA) [15] and has been adapted for practical use in dental implants [16]. However, M-RFA requires a miniature magnet to be placed in the evaluation implant to respond to magnetic pulses, making it difficult to use in orthopedic surgery. In addition, although attempts have been devoted toward diagnosing the installation strength of orthopedic implants using M-RFA, the excitation force remains poor. The evaluation of orthopedic implant stability was possible only when a strong magnet was fixed using an adhesive [17]. Therefore, the use of a laser pulse as an excitation source that impacts the orthopedic implant is necessary to diagnose stability through RFA.

In conventional L-RFA, the impact force of the laser-induced vibration is the reaction force of the plume eruption generated by laser ablation. Impact dynamics using laser ablation have been studied because the energy conversion process from light energy to kinetic energy is essential for optimizing the L-RFA [18,19]. The vibration induced by laser ablation caused slight damage to the crater and debris on the surface; this is undesirable for medical applications in orthopedic implant installation surgery because it causes damage to the implant surface and antibacterial coating. In addition, the requirement of a high-power pulsed laser system to generate ablation results in increased costs and inconvenience due to high-energy large equipment, such as wearing laser safety glasses. To solve the above issue, natural vibration measurements in L-RFAs using a sweep pulse excitation (SPE) method with low-energy laser pulses were reported [20]. In conventional L-RFA, impulse excitation is performed to induce vibration over a wide frequency range by irradiating a nanosecond high-energy single-shot laser pulse. Contrarily, in the SPE method, the repetition frequency of low-energy laser pulses is swept and varied to detect enhanced vibration at frequencies that coincide with the natural vibration; this corresponds to the discrete measurement of the vibration frequencies obtained from single-shot excitation (e.g., continuous spectrum) by sweeping the measurement of the laser repetition frequency. Since this technique is based on the principle of superimposing photoacoustic elastic waves caused by laser irradiation, it is possible to measure the laser energy density below the laser ablation threshold; if the measurement can be carried out with power that does not require laser safety glasses, it could be an innovative technology for medical applications.

In this study, we evaluated the dependence of the laser irradiation conditions, such as the pulse width, spot size, and energy, on copper and brass disk samples that have similar thermal and mechanical properties to determine the vibrational excitation of spatiotemporal dynamics for realizing efficient evaluations. The clarified dominant dynamics contributed to demonstrating the SPE method’s evaluation by a few mW output powers at a commercial laser pointer level.

## 2. Materials and Methods

**Figure 1:**
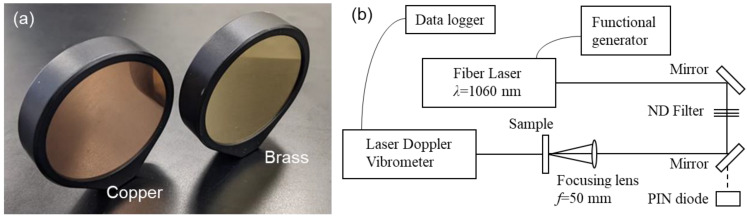
Experimental setup: (**a**) evaluated samples; (**b**) testing layout.

As shown in Figure 1, we used a simple sample and experimental system to evaluate the influence of the laser parameters on the SPE method based on photoacoustic elastic waves. We used two types of simple metal disks with similar thermal and mechanical properties as the evaluation samples: copper and brass. The metal disks were 50 mm in diameter and 1 mm thick and were held in place by a holder approximately 1 mm wide in the circumferential direction, as shown in Figure 1a. Table 1 lists the thermal, mechanical, and optical properties of copper and brass. We referred the optical absorption coefficients at a wavelength of 1053 nm [21].

Figure 1b shows the experimental setup. We used an Nd-doped fiber laser (1060 nm wavelength) that can adjust the arbitrarily variable repetition rate and pulse width to perform the SPE. We used a square-wave voltage generated by a functional generator with stepwise frequency sweeping as an external trigger signal for the fiber laser. We used two mirrors to guide the output laser pulse and ND filters to adjust the irradiation pulse energy. The laser pulse was focused using a lens with a focal length of 50 mm and irradiated at the center of the experimental sample. We monitored the pulse width of the fiber laser using the light transmitted from the mirror, which has a reflectance of 99.5%, with a PIN photodiode. The PIN diode has a wide, undoped intrinsic semiconductor region (I) between a p-type semiconductor (P) and an n-type semiconductor (N) region. Vibration measurements were performed by irradiating a laser Doppler vibrometer at the center of the back surface and measuring the vibration induced by the swept laser pulse irradiated on the opposite side. The laser Doppler vibrometer (VibroOne, polytec GmbH, Baden-Württemberg, Germany) outputs the velocity information obtained by the Doppler effect as a voltage signal, calculates the velocity-based information as phase information, and outputs a voltage signal of the displacement amount. The wavelength and output power of the laser Doppler vibrometer were 633 nm and less than 1 mW, respectively. In this study, we measured the vibration caused by the SPE method and simultaneously utilized the amount of displacement for verification. We recorded the voltage signal output from the laser Doppler meter using a data logger, and the obtained time-series data were used for analysis.

## 3. Distinctions of Sweep Pulse Excitation

In a previous study on the SPE method, an acceleration sensor was used for evaluation, and it was reported that a vibration spectrum waveform similar to that of laser ablation could be obtained [20]. It was also reported that similar vibration spectra were obtained for laser ablation and physical pendulum excitation [19]. Based on these results, similar vibration spectra should be obtained based on velocity or displacement and for the physical and SPE methods. Previous studies [19,20] have demonstrated that different materials (concrete and stainless steel) can be evaluated for natural vibration by a laser with different geometries (rectangular block and bolt shape). This indicates that the natural vibration modes obtained with the impulse hammer can be obtained using the SPE method, regardless of the material or size of the evaluation sample. The only difference in laser excitation versus physical excitation has been shown to be that higher-order vibration modes are easier to excite [19]. However, previous studies [20] have not investigated a presence of an induced higher-order vibration mode in the SPE method. In this section, we use copper samples to verify the vibration-induced phenomenon in the SPE method, including a higher-order vibration mode.

**Figure 2:**
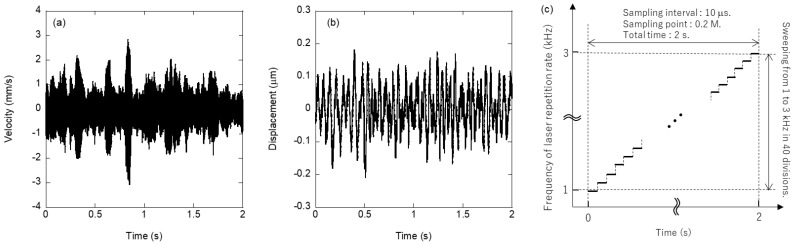
Time−domain vibrational signal for (**a**) the velocity and (**b**) the displacement of a copper disk sample by SPE method using (**c**) stepwise frequency sweeping.

Figure 2 shows the time-series waveform of (a) the velocity signal and (b) the displacement signal obtained from the copper disk when the laser energy was 50 µJ, and the pulse width was 270 ns, sweeping from 1 to 3 kHz in 40 divisions with a sampling interval of 10 us and 0.2 Mpoints, as shown in Figure 2c. The repetition rate of the fiber laser to obtain the induced vibration was swept with a step number of 40. Each voltage signal value was converted to velocity and displacement using pre-calibrated values. As shown in Figure 2a, the velocity waveform indicates that the velocity signal increases when the excitation frequency matches the natural frequency that tends to cause vibrations at each measurement time. However, the displacement waveform shown in Figure 2b could not detect a time waveform similar to the velocity waveform in Figure 2a; this can be attributed to two factors. First, the amount of displacement obtained by laser excitation is in the sub-micrometer order; therefore, although laser Doppler vibrometers have a resolution of several nanometers, a large signal-to-noise ratio cannot be obtained in measurement environments that are not equipped with vibration isolation. This phenomenon was observed not only for the copper samples shown in Figure 2a,b, but also for the brass samples. Although the above problem can be solved by improving the measurement environment, it is not realistic for medical applications considering actual usage conditions, such as operating rooms at clinical sites. Second, there are superimposed vibrations other than a single natural frequency, including environmental noise, which indicates complex displacement behavior rather than a simple one-frequency vibration; this becomes clear when expressed in the vibration spectrum, a frequency space, by a fast Fourier transform (FFT). This section may be divided by subheadings. It should provide a concise and precise description of the experimental results, their interpretation, and the experimental conclusions that can be drawn.

**Figure 3:**
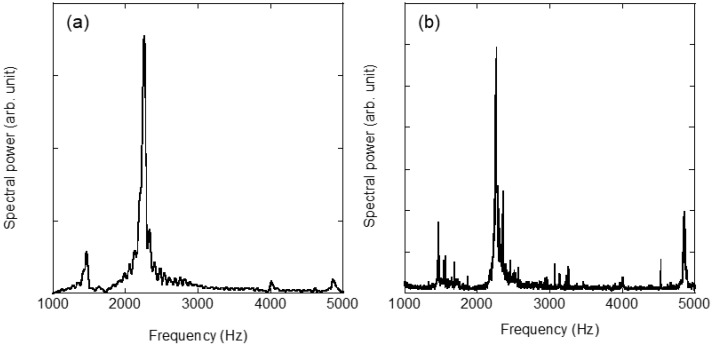
Vibrational spectra of the copper disk sample induced by (**a**) impulse hammer and (**b**) sweep pulse excitation.

**Figure 4:**
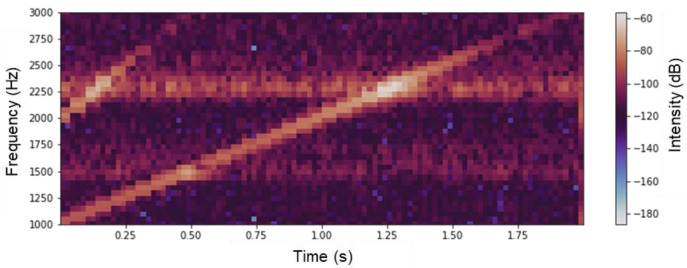
Spectrogram of the vibration of copper disk evaluated from Figure 2a.

Figure 3 shows the vibration spectra of the copper disk evaluated by (a) the impulse hammer and (b) the SPE methods, which are the results of the FFT analysis. Figure 3b shows the FFT analysis of the velocity data shown in Figure 2a. As shown in Figure 3a, the highest natural frequency peak was observed at 2254 Hz; however, other frequency peaks were also observed, indicating a mixture of the vibration modes. The SPE method, shown in Figure 3b, produces narrow, spike-like vibration spectra, as compared with the impulse excitation, because it entails a step-like frequency sweeping that inherently generates discrete vibration data. The highest frequency peak in Figure 3b is located at 2262 Hz, which is different from the measurement with the impulse hammer; however, it is within the error range because the sweeping frequency range of 2 kHz is swept at 40 steps, which is less than the resolution of the swept frequency (i.e., 50 Hz/step). Therefore, in the evaluation using the SPE method, it is more preferable to utilize the velocity and acceleration data shown in Figure 2a and previous studies [20] than the displacement data shown in Figure 2b, from a practical perspective. The two graphs in Figure 3a,b are similar, and it is evident that the SPE method has the same performance as the physical excitation and single excitation using a laser. Previous studies have reported higher-order vibrational mode excitation in a single excitation using laser ablation, which is more efficient than physical impacts [19]. As shown in Figure 3b, vibrations above 3 kHz, outside the sweeping frequency range in the SPE method, were observed in the peak at 4856 Hz. However, the irradiated laser pulse energy was lower than that of the ablation threshold, and it was considered difficult to excite vibration modes other than the excitation frequency using the SPE method. To reveal excitation dynamics using the SPE method, Figure 4 shows the time-series vibration waveform spectrogram depicted in Figure 2a. The spectrogram is the result of passing the composite signal through a window function and calculating the frequency spectrum, which is represented by a three-dimensional graph (time, frequency, and strength of the signal components). As the excitation laser repetition frequency was swept, a line of forced vibration occurred from 1000 to 3000 Hz, and, simultaneously, harmonic excitation starting at 2000 Hz was observed. Since a rectangular-wave voltage containing only odd-order harmonics is used to sweep the excitation laser, even-order harmonics are unlikely to appear. Therefore, harmonic distortion associated with the rattling of the fixation of the evaluation sample is considered to have occurred. When using the SPE method, it is necessary to consider in advance that excitation at the integer multiples of the irradiation frequency may occur due to the harmonic distortion originating from the evaluation sample. The SPE method performs active excitation at multiple excitation frequencies within a short period and indicates complex vibration behaviors, where harmonic and forced excitations are superimposed. However, because the forced excitation intensity is extremely small (−30 dB at resonance) due to the low-energy pulses, it is possible to perform measurements with a high signal-to-noise ratio.

## 4. Results of Dependence Evaluations and Discussion

The SPE method at a low-energy laser pulse enables an active excitation equivalent to an impulse hammer or laser ablation. To take full advantage of the SPE method, the physical principles of the laser-induced excitation phenomenon must be understood and optimized by considering the spatiotemporal dynamics. Thus, we evaluated the dependence of the vibration intensity on the pulse width to clarify the temporal dynamics of the laser-induced vibration in the SPE method. We evaluated the irradiation spot-size dependence to reveal the spatial dynamics. Based on the results of this evaluation, we determined the other parameters required for the impact laser system to realize a highly efficient SPE method in the investigation of the irradiation power dependence.

### 4.1. Pulse-Width Dependence

In physical excitations, such as impulse hammering, the excitation time width is an essential parameter for determining the possible frequency range that can induce vibration. Therefore, in conventional L-RFA using laser ablation with a high-energy laser pulse, nanosecond pulses have the advantage of stable impulse excitation in the ultrasonic range. Nanosecond laser pulse width might be unnecessary in the case of the SPE method because low-energy laser pulses can be excited only at an adjusted frequency. However, it takes advantage of the photoacoustic elastic waves resulting from the absorption of laser energy. It is necessary to consider the photoacoustic conversion (i.e., electron–lattice interaction) that occurs in the order of nanoseconds. Therefore, the temporal dynamics associated with thermal diffusion may affect the magnitude of the excitation. Thus, we investigated the dynamics of the heat generation and dispersion by varying the pulse width of the irradiation laser pulse in the SPE method.

**Figure 5:**
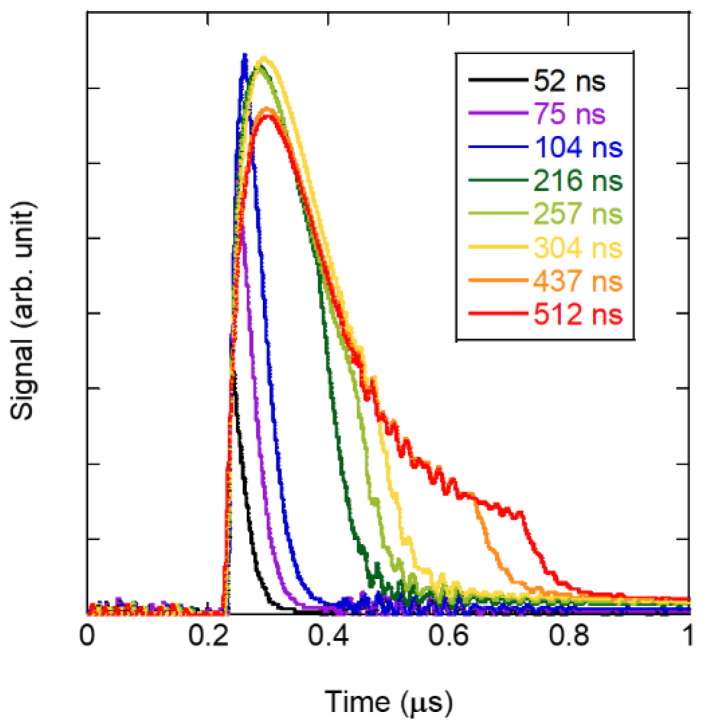
Variation of the impact laser pulse at different pulse-width settings.

Figure 5 shows the variations of the irradiation laser pulses at different pulse widths given by the PIN photodiode shown in Figure 1. We adjusted the pulse width of the fiber laser used for excitation by controlling the current of the pump laser diode that excites the laser medium; thus, the pulse waveform has a tail in the latter part. In this study, the pulse width was defined as the time interval, i.e., 1/e^2^ of the peak signal in Figure 5. The pulse-width variation was characterized by a slight decrease in peak kurtosis at pulse widths wider than 100 ns. A distinct tail pulse appeared at pulse widths wider than 250 ns, and the pulse width was constant at 210 ns when defined by the full width at half maximum. When evaluating pulse-width dependence, since the pulse energy changes as the pulse width changes, we used ND filters to adjust the energy to be constant regardless of the pulse width.

**Figure 6:**
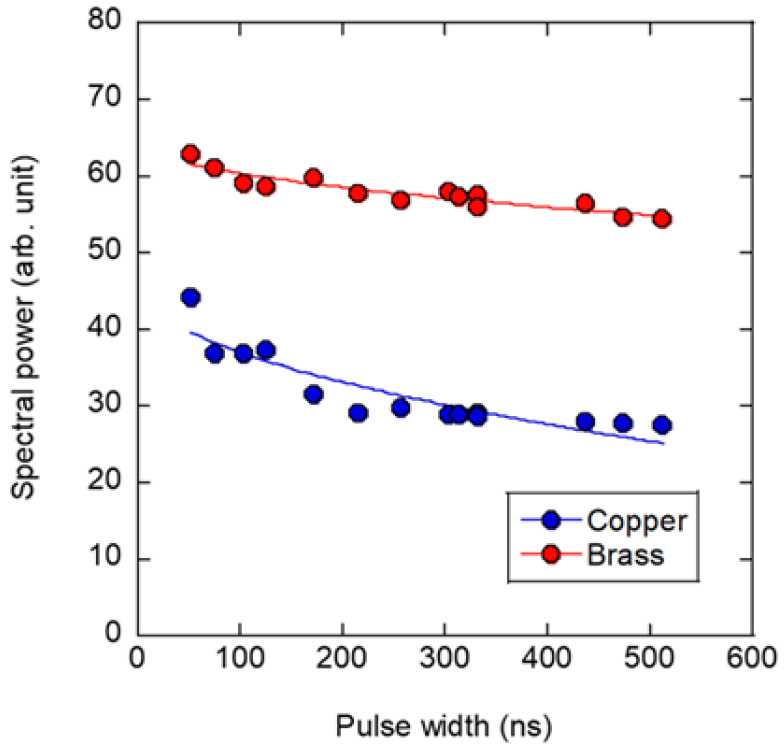
Pulse-width dependence of vibrational power induced by single-frequency low-energy laser excitation.

Figure 6 shows the results of the pulse-width dependence of the spectral power induced by the SPE method. The value on the vertical axis indicates the vibration spectral power *P* when the experimental sample was subjected to vibration at the same repetition frequency as the natural frequency observed with the impulse hammer, as shown in Figure 3a. We adjusted the repetition frequency of the SPE method to 2240 Hz for the copper disk and 2120 Hz for the brass disk. The frequencies were theoretically consistent wherein copper and brass exhibited close natural frequencies because these frequencies were determined proportional to the mechanical property parameter (*E*/*ρ*)^1/2^, where *E* denotes Young’s modulus and *ρ* is density. The pulse energy was fixed at 18 μJ using ND filters. The spectral power of the laser-induced vibration decreased with an increase in the pulse width. Pulse-width dependences, which were discussed in the interaction of nanosecond laser pulses with materials, are often explained through thermal diffusion. For example, a scaling law was reported for the laser-induced damage threshold (ablation threshold) caused by intense nanosecond laser pulses, whose dependence on the pulse width τ is proportional to *τ*^1/2^ [22,23]. This can be explained by the coefficient *τ*^1/2^ in the diffusion equation because the heat storage produced by the absorption of laser energy is significant for its interaction with the irradiated material. The fittings in Figure 6 are the results for *P*∝*τ*^1/2^. In this study, we obtained the same scaling law as in the previous study. A comparison of the copper and brass fittings shows that the brass slope was sufficiently smaller than the copper slope because of the difference in the thermal diffusion coefficient, i.e., thermal conductivity. We had assumed that copper, which conducts heat better and is more prone to diffusion and more sensitive to pulse-width fluctuations. However, brass has a small range of variation. Both materials were vibrated at approximately 2200 Hz. The time interval of the laser pulse was approximately 450 μs, which was 103 times longer than the laser pulse width and provided sufficient time for heat diffusion. Therefore, the heat accumulation effect of repeated pulse irradiation was considered minimal and was determined by the dynamics of a single pulse, which was completed by the next pulse irradiation.

### 4.2. Spot-Size Dependence

**Figure 7:**
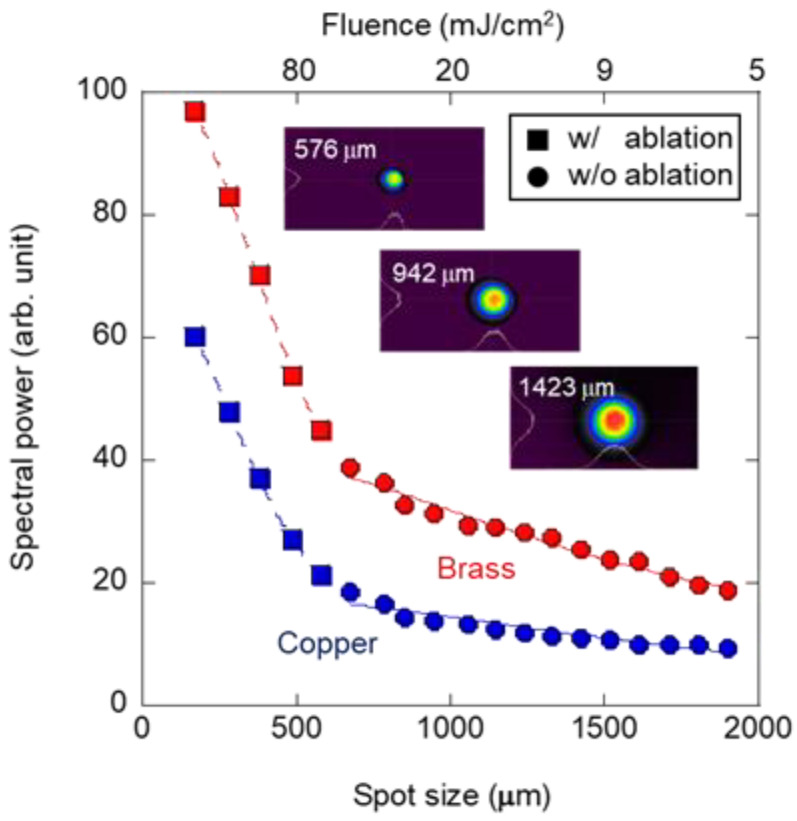
Spot-size dependence of vibrational power induced by single-frequency low-energy laser excitation.

Evaluating the pulse-width dependence revealed that thermal diffusion affects laser excitation through the SPE method. The spatial dynamics due to the generation of thermal gradient caused by the spot size of the laser irradiation are significant, in addition to the temporal dynamics, due to the pulse width over time of the thermal diffusion. Therefore, in this study, excitation was performed at different spot sizes to evaluate the dependence of an intensity of the laser-induced vibration, i.e., spectral power. The spot size was controlled by adjusting the installation position of the focusing lens toward the in-focus side. We measured the laser spot size by installing a CMOS camera at the evaluation sample position, defined as 1/e^2^ of the Gaussian profile. Figure 7 shows the spot-size dependence of the vibrational power induced by single-frequency low-energy laser excitation. The horizontal axis shows the average spot size calculated in the horizontal and vertical directions, and the vertical axis represents the vibration intensity at single-frequency irradiation, which is the same as the vertical axis in Figure 6. We adjusted the laser pulse energy to 157 μJ. As shown in Figure 7, the focused profiles of the Gaussian distribution were maintained, regardless of the spot size during the dependence evaluation. The two types of slopes for both the copper and brass samples appeared definitely, as shown in Figure 7. This is because laser-induced ablation occurs when the energy fluence exceeds the threshold (approximately 80 mJ/cm^2^) for sufficiently small spot sizes. We performed the fitting, as shown in Figure 7, using *P*∝exp(−*d*^2^). We can obtain this relationship using the thermal-diffusion equation. Ablation dramatically increases the intensity of vibrations because of the enhanced excitation force associated with the ablation plume generation. However, for pulse energies below the damage threshold, the slope is less because the vibration is only associated with photoacoustic elastic waves. The smaller the spot size, the stronger the laser-induced vibration that can be observed with or without laser ablation. This is attributed to the large thermal gradient generated by the local concentration of the heating area. Generally, the laser spot size is proportional to the focal length of the focusing lens, the wavelength of laser pulse, and is inversely proportional to the laser incident diameter of the focusing lens. Designing a focusing system with a smaller spot size contributes to an efficient SPE method. The samples used in this study had a finite diameter of 50 mm. The spot-size dependence is influenced by the aspect ratio of the laser to the spot size. In the case of the disk with a fixed circumferential direction used in this study, assuming a simple natural vibration that excites the center of the disk in the plane direction, excitation by a laser spot with the same diameter as the disk or larger will be inefficient, and it is estimated that the size of the spot should be efficient up to approximately half the diameter of the disk.

### 4.3. Irradiation Power Dependence

**Figure 8:**
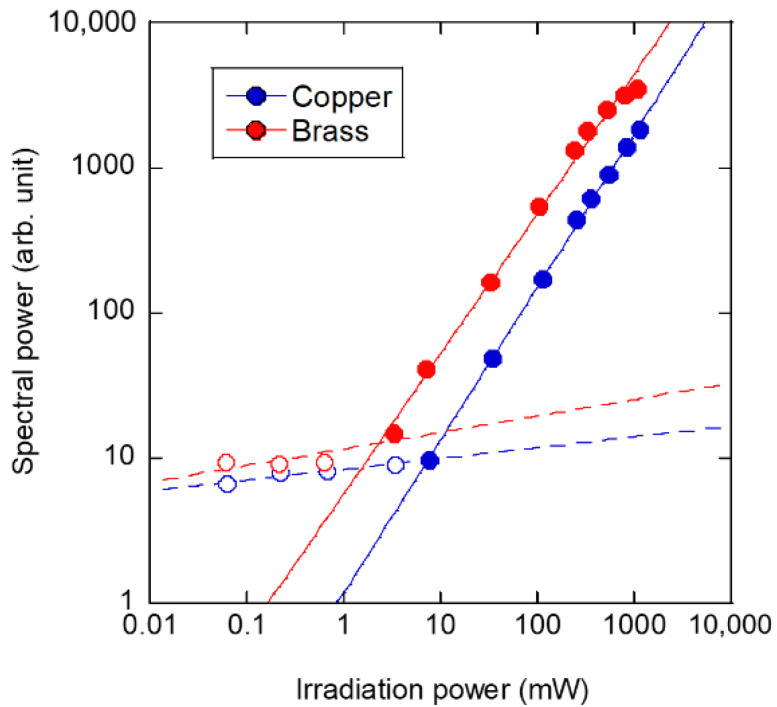
Irradiation power dependence of spectral power in characteristic vibration.

We evaluated the dependencies of the spatiotemporal dynamics in Section 4.1 and Section 4.2. As a result, it was evident that shorter pulse widths and smaller spot sizes were better in the SPE method due to the heat diffusion phenomena. Finally, we evaluated the performance at low power, which is the greatest advantage of the SPE method. Figure 8 shows the irradiation power dependence of the vibration intensity on the copper and brass disks induced by a single laser repetition rate. During the evaluation, we fixed the laser pulse width and average spot size calculated with the vertical and horizontal diameters at 270 ns and 170 μm, respectively, and we set the repetition rate to 2240 Hz (copper disk) and 2120 Hz (brass disk), which are natural frequencies. The horizontal axis of Figure 8 shows the irradiation laser power calculated as the product of the pulse energy and repetition frequency, and the vertical axis shows the vibration spectral power during excitation. As a result of the evaluation, two power approximations, modeled *y* = *xa* (where *a* is a fitting parameter), were used to fit at lower and higher irradiation power ranges as solid and dotted lines in Figure 8. The fittings at higher irradiation powers showed an almost linear relationship (*a* = 1), i.e., *a* = 1.05 in copper, and *a* = 0.97 in brass. However, the fittings at lower irradiation power ranges were almost constant (*a* = 0), i.e., *a* = 0.074 in copper, and *a* = 0.11 in brass. The constant relationships with irradiation power meant that it appeared to be a local mode and/or background noise level. The thresholds that could enhance the laser-induced vibration by the resonance effect were calculated as the intersection point of the regression expressions. Enhancing the vibrations above the background level was confirmed at threshold values of approximately 7.41 mW for the copper disk and approximately 2.39 mW for the brass disk. In the brass, there was a slight saturation of the spectral power obtained from laser-induced vibration at maximum power, indicating that a sufficient excitation force is obtained. The difference in the vibration intensity presented by the spectral power and threshold, which can enhance the effect between copper and brass, was approximately twice as large for Figure 6, Figure 7 and Figure 8, which corresponds to the difference in the laser absorptions shown in Table 1. Since the laser-induced vibration amplification due to the resonance effect was linearly correlated, as shown in Figure 8, the optical absorption was considered to be the difference in the excitation intensity presented by the spectral power. Therefore, to achieve highly efficient vibration excitation utilizing the resonance effect and effective SPE method, it is essential to suppress the temporal thermal diffusion by shortening the pulse width, generating a local thermal gradient by reducing the spot size and selecting a laser wavelength with high absorption for the evaluated material. In the evaluation using the SPE method, natural frequencies are easier to measure if the evaluated material has a low damping coefficient and high spring constant proportional to Young’s modulus. Orthopedic implants are typically made from titanium alloys (Ti-6Al-4V), which have a Young’s modulus of 110 GPa, comparable to copper and brass. Therefore, the SPE method can be used to evaluate the stability of orthopedic implants. In addition, it can be applied to a wide range of metallic materials because the fastening strength of stainless-steel bolts, which has a higher Young’s modulus of 200 GPa, was adopted and has already been demonstrated in the SPE method [20].

## 5. Conclusions

In this study, we aimed to clarify the influence of laser irradiation parameters on different metal disk samples to investigate the dynamics of the SPE method. In the SPE method, the photoacoustic elastic wave is enhanced through the resonance effect to realize an ultralow-power L-RFA laser diagnosis scheme. We evaluated the temporal dynamics using the pulse-width dependence, spatial dynamics using the spot-size dependence, and linearity with respect to excitation power using irradiation power dependence. The SPE method can explain the heat dispersion phenomenon. The results revealed that for a highly efficient SPE scheme utilizing the resonance effect, the pulse width was shortened, the spot size was reduced, and a laser wavelength with a high absorption coefficient was selected.

In LRS based on L-RFA, the classification of the laser system by the International Electrotechnical Commission is significant for convenience. The irradiation power threshold for enhancing the photoacoustic elastic wave using the resonance effect obtained in this study was less than 5 mW. By decreasing the intensity of the laser pulse through selecting a laser wavelength with a higher absorption coefficient for the evaluated material, we can realize L-RFA with the SPE method at lower power. This will reduce the cost of the LRS system in addition to increasing the convenience of the inspector. In conclusion, the achievement of the SPE method, which utilizes the enhancement of photoacoustic waves through the resonance effect obtained in this study, can be a milestone for LRS in a wide range of fields. The contribution will not only be useful in the medical field, such as the diagnosis of orthopedic implant stability, but also in the industrial and infrastructure fields.

## Figures and Tables

**Table 1:** Thermal and mechanical properties of copper and brass.

Property		Copper	Brass
Thermal conductivity	(W/mK)	386	106
Specific heat	(J/kgK)	419	386
Thermal expansion coefficient	(/K)	17.7 × 10^−6^	20.8 × 10^−6^
Density	(kg/m^3^)	8960	8400
Young’s modulus	(GPa)	130	106
Poisson ratio		0.34	0.35
Absorption at 1053 nm [21]	(%)	6	12

## Data Availability

Not applicable.
